# A Broad Consideration of Risk Factors in Pediatric Chronic Pain: Where to Go from Here?

**DOI:** 10.3390/children3040038

**Published:** 2016-11-30

**Authors:** Hannah N. McKillop, Gerard A. Banez

**Affiliations:** 1 Case Western Reserve University, 11220 Bellflower Rd, Cleveland, OH 44106, USA; 2 Cleveland Clinic Children’s Hospital for Rehabilitation, Pediatric Pain Rehabilitation Program, CR 11/ 2801 MLK Jr. Drive, Cleveland, OH 44104, USA; banezg@ccf.org

**Keywords:** chronic pain, pediatrics, risk factors, family factors, peer factors, biopsychosocial, development

## Abstract

Pediatric chronic pain is a significant problem associated with substantial functional impairment. A variety of risk factors have been found to be associated with chronic pain in youth. The greatest amount of evidence appears to support that temperament, anxiety, depression, subjective experience of stress, passive coping strategies, sleep problems, other somatic-related problems, and parent and/or family factors are important variables. However, a great deal of this research focuses on a single risk factor or on multiple risk factors in isolation. Much of the literature utilizes older diagnostic criteria and would benefit from replication, larger sample sizes, and comparison across pain disorders. Problems also exist with disagreement across definitions, resulting in inconsistency or unclear use of terms. Furthermore, recent consideration has suggested that outcome measures should include functional disability in addition to pain. A second generation of research is needed to shed light on the complex interactions that likely play a role in the transition from acute to chronic pain. Building on recent calls for changes in research in this area, we propose the next steps for this research, which involve consideration of both biopsychosocial and developmental contexts.

## 1. Introduction

Chronic pain is a significant problem among children and adolescents and is associated with significant functional disability and other poor outcomes, including problems with school attendance and performance, difficulties with peer relationships, disruptions in family life, appetite, sleep, and participation in enjoyable activities [1,2,3]. Children and adolescents with chronic pain also report frequent use of medications, placing the child at risk for future overuse [2]. Although prevalence estimates fluctuate drastically across studies, geographic location, and medical diagnoses, chronic and recurrent pain is thought to be very common in youth [4]. For example, one large-scale international study demonstrated prevalence of headache as 54.1%, stomachache as 49.8%, and backache as 37% [5].

The impact of chronic pain extends beyond the individual as well. Families of children with chronic pain frequently seek professional medical or mental health services [2], which are likely to be very expensive for the family and the healthcare system. With a stronger understanding of the mechanisms that contribute to the development of chronic pain from acute pain, the possibility of developing successful treatment methods could contribute to a reduction in the attendant psychological, social, learning-related, developmental, and monetary costs.

Although there have been many studies of specific risk factors that may contribute to chronic pain, the mechanisms that lead to a progression from acute pain to chronic pain are not well understood. A more nuanced understanding of the complex interactions among a variety of factors has the potential for providing valuable information about the development and maintenance of these conditions, as well as for developing more effective treatments for children in this situation. The focus of this narrative review is on risk factors associated with the most common chronic pain diagnoses seen in pediatric practice.

## 2. Methods

Articles for consideration in this narrative review were identified through a computerized search of electronic databases primarily including PsycINFO, PubMed, and Google Scholar. The search requested all citations that included either a specific chronic pain diagnosis (e.g., complex regional pain syndrome, chronic migraine) or the term “chronic pain” as one identifier and “pediatric,” “children,” “adolescent,” “youth,” “risk factor,” or “predictor” as a second identifier. Diagnoses where pain is the secondary issue (e.g., cancer, cerebral palsy) were not included. Given the broader search capacity of Google Scholar, the first five to ten pages of results (the equivalent of 50–100 articles) were manually scanned for relevant material for each specific search. The search was narrowed to include papers only published within the past 15 years where possible, unless recent research was sparse and/or seminal articles had been published prior to the year 2000.

The literature search yielded a preliminary database of approximately 13,184 published articles. Abstracts were manually scanned for relevance to the review. Approximately 234 articles were obtained for consideration. Articles were then reviewed for inclusion in the review, from which 135 were selected for inclusion in this review.

## 3. Current Status of the Literature on Risk Factors for Pediatric Chronic Pain

The extant literature on pediatric chronic pain has identified a variety of potential risk factors, including demographic factors such as age and sex, factors related to the individual such as coping style, and familial or environmental factors. These studies represent an excellent first generation of research that has helped to identify potential risk factors for further study. Interestingly, many of the risk factors identified were predicted in an early survey study comparing children with recurrent abdominal pain to children without these symptoms from the 1950s, including family history of pain, temperament, and anxiety symptoms, as well as higher incidence in females and onset during adolescence [6]. However, it is important to move forward to a next stage of research to better understand the role of these factors in the mechanisms that lead acute pain to become chronic, as well as contribute to the maintenance of chronic pain.

The literature at present has often focused on one medical chronic pain diagnosis at a time, which is useful in the understanding of that particular disorder but is difficult to generalize to chronic pain diagnoses in general. In addition, issues with diagnostic criteria have been raised with the implementation of the fifth edition of the Diagnostic and Statistical Manual of Mental Disorders (DSM) [7]. Many of the studies also have small sample sizes (e.g., often ranging from 15–35 participants), inconsistent definitions or use of unclear terms (e.g., variation across studies in terms of the definition of headache), and static forms of measurement (e.g., self-report questionnaire). Few studies examine the longitudinal impact. In addition, studies often focus on a single risk factor or explore multiple risk factors in isolation. This type of research design, while necessary for the original identification of these risk factors, cannot provide information about causality or the complex interactions among variables that may explain a larger portion of the variance in chronic pain.

**Figure 1:**
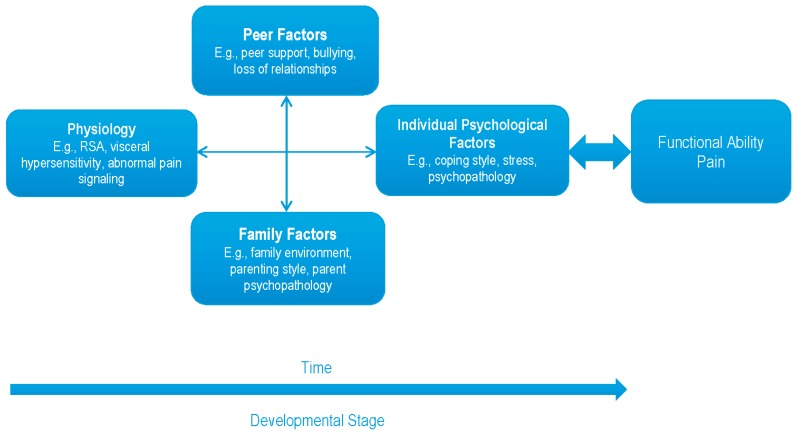
Incorporation of biopsychosocial factors into a developmental framework. RSA: respiratory sinus arrhythmia.

This article first provides an overview of the current state of this ”first generation” of research, which can serve as a starting point for consideration of potential variables to be included in more complex models. In line with this idea, we next identify studies that embody the second generation of research, and then propose next steps to continue in this direction (see Figure 1). These next steps consider the development of chronic pain from acute pain, taking into account: (1) developmental stages; (2) family and peer factors; (3) physiological components; and (4) will integrate the interplay of multiple risk factors. It is proposed that functioning represents as much importance in outcome as the presence of pain.

### 3.1. A Review of Risk Factors

A large number of risk factors of pediatric pain have been studied, and this literature spans a variety of categories. Many studies have focused on within-person factors, that is, factors specific to the child himself or herself, such as temperament and coping style. Between-person factors, such as parent psychopathology and social functioning, are considered. Demographic factors are also included, such as sex, age, and ethnicity.

### 3.2. Within-Person Factors

#### 3.2.1. Demographic Factors

Briefly, several demographic factors are considered in relation to pediatric chronic pain, including age, sex, and ethnicity.

Increased age (typically early adolescence rather than early childhood) is associated with various pediatric chronic pain disorders, including low back pain [8] and musculoskeletal pain [9]. That is, chronic pain appears to most often occur in adolescence rather than earlier in childhood. However, recurrent abdominal pain appears to peak below 5 years of age and again between ages 8–10 years [10]. This particular finding speaks to the potential relationship between pain and developmental stage, which will be discussed later in this article.

The literature suggests that female youth report more severe headache and back pain [11,12,13,14], chronic musculoskeletal pain [9], temporomandibular pain disorders [15], and recurrent or functional abdominal pain than male youth [16]. However, some studies have found only minimal support for this relationship, demonstrating small effect sizes for duration and intensity of pain for girls versus boys [17].

A sex by age relationship has been observed in children and adolescents with chronic migraine. Based on the extant literature, chronic migraines appear to be more common in boys than girls prior to puberty [18]. Thus, onset of migraines in boys is likely earlier than for girls. This estimate changes by the onset of puberty, and the prevalence of chronic migraines in girls is estimated to double that of boys at that stage [18].

Race and ethnicity have rarely been the sole focus of research on pediatric chronic pain disorders, although it is well studied in adults. Briefly, studies have found the following: (1) participants of European descent were more likely to have juvenile idiopathic arthritis than children of other descent [19]; (2) American Indian adolescents had the highest rate of recurrent headaches followed by white adolescents [20], and Asian and Pacific Island adolescents were the least likely to experience recurrent headaches [20]; (3) African American youths were more likely to experience a variety of pains related to temporomandibular joint disorders than their Caucasian counterparts [21]; (4) Hispanic ethnicity was associated with higher widespread pain scores in children with acute pain, presurgical, and chronic pain [22]; and (5) Jewish children experienced significantly more headaches than Arab children in a sample from Northern Israel [23]. Other groups have found no significant differences among ethnic groups in samples of children with a variety of chronic pain diagnoses [24,25]. In addition, ethnicity did not emerge as a risk factor for disability in a study of children with chronic back pain [26].

In sum, demographic factors including age, sex, and ethnicity evidence some support of potential risk factors for pediatric chronic pain. These variables warrant continued research in their potential as risk factors for youth. Studies that assess demographic factors in combination with other identified variables are important for improving our understanding of risk pathways.

#### 3.2.2. Temperament

Broadly defined temperamental factors have been cited in the literature as potential risk factors [27]. For example, infants who were more active and struggled to develop important routines were more likely to develop recurrent abdominal pain later in childhood [28]. Children with recurrent abdominal pain were more “temperamentally difficult” than those without pain, such that girls had more of an irregular temperament style and boys were more likely to withdraw in novel situations [29].

In a study of children with juvenile primary fibromyalgia syndrome, temperament was described as a combination between mood, daily habits, and attentional abilities [30]. The study demonstrated that these children displayed lower mood, irregularity of daily habits, lower task orientation, and higher distractibility than a comparison group (participants had arthritis) as well as a control group [30]. Thus, a difficult temperament may represent a mechanism by which acute pain becomes chronic and/or plays a role in maintenance of impairing pain. Temperament may also influence what eventually becomes a child’s coping style [31], which could have important implications for the way a child responds to prolonged experience with pain. While temperament itself is not modifiable by definition, if high-risk temperamental styles are identified early on in children with pain, the first targets of intervention could focus on the development of adaptive coping strategies before pain becomes chronic. However, other factors, outlined below, may represent more promising avenues for prevention and early intervention efforts.

#### 3.2.3. Psychological Disorders

Chronic pain has been found to be associated with a variety of psychological issues, including anxiety, depression, anger, conduct problems, and mental health issues in general.

*Anxiety*. Anxiety has been frequently studied as a risk factor for numerous chronic pain disorders. While the issue of causality is often cited as a problem with this area of research, a recent study demonstrated a strong temporal association, with anxiety disorders preceding reports of chronic back/neck pain, headaches, and “any chronic pain” [32]. However, another study of predictive factors for recurrent abdominal pain in children specifically did not find such a temporal relationships [33].

Cross-sectional studies do appear to show a somewhat consistent relationship between anxiety and chronic pain. Anxiety has been significantly associated with general musculoskeletal pain for girls [34], migraine with aura [35], and recurrent abdominal pain [36,37]. A recent study supported this finding in a general chronic pain sample and demonstrated that youth with abdominal pain reported higher overall anxiety as well as more panic-somatic symptoms relative to other pain groups [38]. Other studies demonstrate no relationship between anxiety and pain, including studies of juvenile idiopathic arthritis [39] and headache or abdominal pain [40].

Sensitivity to anxiety has also been posited as a factor that contributes to the maintenance of postsurgical pain in children and adolescents [41]. Anxiety sensitivity is defined in terms of the degree to which an individual interprets or predicts anxiety symptoms as being related to significantly harmful somatic, psychological, and/or social outcomes [41], and was the only predictor of maintenance of or recovery from moderate/severe chronic postsurgical pain 12 months after the surgery [41]. Anxiety, when it becomes impairing, could be considered a maladaptive response to pain, if that is indeed the order of occurrence. Longitudinal studies could lend predictive power to the current understanding of the chronicity of pain and the role of anxiety.

*Depression*. Depression is strongly related to anxiety [42] and is worth examination in relation to chronic pain in its own right. Depression appears to be an important factor in pediatric chronic pain disorders. As with anxiety, the question of whether we can infer causality between depression and pain is often cited. However, a temporal relationship was found between preceding depression diagnoses and headaches or “any chronic pain” [32]. Another study explored childhood predictors of abdominal pain in adolescents over the course of 13 years at six different time points and found that the presence of depressive symptoms in childhood (at age 12) predicted recurrent abdominal pain two years later [16]. This finding suggests that depression may play a role in the transition from acute to chronic pain. Cross-sectional studies have also demonstrated associations between depression and irritable bowel syndrome [43], as well as with chronic daily headache when compared to control samples [35].

Two studies used nationally representative community samples to understand the connection between chronic pain and psychopathology in youth who are not receiving treatment for chronic pain. First, an association was found between musculoskeletal pains and depression in both boys and girls [34]. A second study found that 16% of all adolescents are at risk for developing depression, but this risk increases to 45% when adolescents have daily pain [44]. Studies with larger sample sizes in pediatric clinical populations are needed to further our understanding of the interplay between depression, anxiety, and pain.

*Other disorders and mood problems*. A small body of literature emphasizes a connection between trauma and chronic pain, primarily focusing on abuse (physical or sexual) or injury (e.g., sports injury, accident) [45]. These studies suggest that early posttraumatic stress disorder (PTSD) or trauma-related symptoms predict later functional impairment and pain [45]. However, more research is needed to understand the connection between trauma and pain in the pediatric population.

Other studies have highlighted issues related to anger [46], oppositional defiant disorder and attention-deficit hyperactivity disorder [34], conduct problems, [47] and a broader “psychopathology” variable [11,48,49,50]. Furthermore, “negative emotions” have been identified as a risk factor of moderate quality for headache in youth [13]. Broad terms such as “psychopathology” and “negative emotions” are likely too vague and difficult to replicate, and they may not be particularly useful in a clinical setting. Longitudinal studies will be particularly useful in guiding our understanding of the role of psychological factors in the development of chronic pain.

#### 3.2.4. Stress

The subjective experience of stress demonstrates a strong relationship with pain. One study found that perceived stress explained a significant amount of the variance in present and worst pain intensity for younger children [25]. For adolescents, perceived stress was associated with present pain intensity only [25]. These findings are consistent with the adult literature on perceived stress and pain.

Houle and Nash [51] posit that stress is a risk factor in the “chronification” of headache in adults through several mechanisms, including daily stressors and chronic hyperarousal. Stress is also posited to indirectly relate to a series of other potential variables that can impact pain, such as fear of pain, locus of control, dysregulation of sleep and eating routines, overuse of medications, and psychopathology [51]. Thus, stress may play a crucial role in both the etiology and maintenance of pain problems. Finally, the experience of negative or stressful life events appears to be related to chronic pain [16,52,53,54]. Future research is needed to understand these complex interactions among stress and other variables as they relate to pain, especially in the pediatric population.

#### 3.2.5. Coping Style

Compas et al. [55] define coping as “conscious volitional efforts to regulate emotion, cognition, behavior, physiology, and the environment in response to stressful events or circumstances”. This widely accepted definition, especially as it relates to children and adolescents, takes developmental level into account, stating that an individual’s development might facilitate or hinder the type of coping strategy that is available to and/or used by the individual [55]. Furthermore, unlike adults, children may not have developed a fully formed “coping style” or approach that they typically rely upon [26]. Children and adolescents may be more likely to employ a larger variety of coping strategies than adults as they attempt to form their own individual coping style. Developmental stage, therefore, is important to take into consideration when designing studies. Some strategies are seen as positive or adaptive and others are seen as maladaptive.

In a study of pediatric pain patients, coping was correlated with depression and disability [56]. The most common strategies were broken down into maladaptive (internalizing and catastrophizing) and adaptive (problem-solving and behavioral distraction) [56]. A strong relationship was found between the maladaptive coping strategies (e.g., dependency, denial, catastrophizing) and chronic pain for adolescents, and is consistent with findings in the adult literature [57,58,59,60]. Differences in coping strategies across diagnoses have also been found [56]. A musculoskeletal group reported greater disability and more difficulty coping than the headache group [56]. Further support relates pain catastrophizing to pediatric chronic pain conditions [61]. Together, these findings suggest that coping strategies represent an important area of focus for prevention and intervention strategies for chronic pain. Some promising studies, which are reviewed in depth below, have utilized more sophisticated methodology in order to improve our understanding of the role of different coping strategies.

#### 3.2.6. Fear, Avoidance, and Beliefs

Asmundson et al. [62] argue that fear, anxiety, and avoidance appear to play a circular role in chronic pain. An original injury or experience of pain may lead to fear, which leads to avoidance of activities that may cause more pain. Avoidance of the anxiety felt in situations where an individual might expect to feel pain may strengthen the behavioral avoidance response as well [63]. Anxiety and avoidance may increase the fear. However, with avoidance of activity, eventual involvement in such activities is likely to involve a great deal of pain. Like a self-fulfilling prophecy, the fear of pain is confirmed, leading to continued avoidance of activities. Thus, Asmundson et al. [62] argue that the paradoxical and cyclical nature of the fear and avoidance relationship in chronic pain is problematic.

This problematic cycle includes fear-avoidance behaviors, which have been cited as potential risk factors for adults in the transition from acute to chronic pain with musculoskeletal pain [64], low back pain [65], and back and neck pain [66], as well as with maintenance of chronic pain [67]. In addition, fear of pain on its own has been identified as a risk factor in youth [61]. Studies such as these need to be replicated in pediatric populations to help understand these pathways in children.

A variety of specific beliefs have also been attributed to poor pain outcomes. In a controlled study of adolescents with recurrent abdominal pain (RAP), participants reported significantly greater concerns about undiagnosed physical disease and greater belief in susceptibility to functional impairment by pain and other physical symptoms [36]. Children with recurrent abdominal pain also appear to have significant hypochondriacal beliefs [36]. In the case of headache, locus of control and self-efficacy appear to be important risk factors [46]. A helplessness–hopelessness factor predicted adjustment to low back pain in adults one year later [68]. Thus, some studies support the idea that different types of negative beliefs about the self are related to chronic pain.

#### 3.2.7. Sleep Problems

Related to psychological well-being, the relationship between pain and sleep is well established. Sleep problems are reported by over half of youth with chronic pain [3,69] and are consistently associated with a variety of pain syndromes, including headache [12], musculoskeletal pain [70], fibromyalgia [71], and pain in general [72]. Relatedly, fatigue is often a comorbid complaint of children with chronic pain, which is likely related to poor sleep [70]. Mood and depression appear to complicate this relationship [3,72], and likely require more sophisticated models to understand the full nature of this multivariate relationship.

#### 3.2.8. Summary

The greatest amount of evidence suggests a number of risk factors in relation to pediatric chronic pain, including difficult temperament, anxiety, depression, subjective experience of stress, passive coping strategies, and sleep problems. Some evidence suggests that fear and avoidance behaviors as well as negative beliefs about the self may be risk factors for the development and/or maintenance of pediatric chronic pain. Mixed results for increased age, female sex, and a general psychopathology variable warrant further investigation. However, as discussed, there are a number of problems with the current literature. Small sample sizes, imprecise terminology, lack of comparison studies, and correlational analyses make it difficult to generalize results to the pediatric pain population as a whole. All studies reviewed primarily utilize pain as their outcome measure as well. A second generation of research is needed to build upon this first generation that incorporates more sophisticated methodology and sound research design, examining functional ability as an additional outcome measure.

### 3.3. Between-Person Factors

Several factors outside of the individual patient have also been found to be influential in pediatric pain, including parent and family variables. Palermo et al. [73] state that there are many gaps in the existing literature on family and parent influences in pediatric chronic pain, and that longitudinal studies are needed to understand the family impact on the development of a child’s chronic pain. This section provides a brief overview of the current research on between-person risk factors, providing a context for the proposed next steps in research.

#### 3.3.1. Parental Psychopathology

Depression and anxiety disorders among mothers and fathers are particularly prevalent for parents of children with chronic pain [16,28,74,75,76]. Thus, this is likely an important consideration in the context of chronic pain. In fact, in a sample of preschool children, maternal depression was the only significant factor of a variety of psychosocial stressors and demographic factors that were examined in association with pediatric chronic pain [76]. Mothers of children with recurrent abdominal pain were significantly more likely than mothers of healthy controls to have a lifetime history of and current anxiety, depressive, and somatoform disorders and poorer overall quality of life [77]. A “parent distress” distress also appears to impact youth chronic pain [78]. Notably, little research on fathers has been conducted in this population [73]. Finally, Palermo et al. [73] point to research suggesting that maternal distress and child chronic pain are bidirectional in nature, but this dynamic needs to be examined further. While some interesting associations have been found, it is clear that more research is needed to understand the role of each of these factors in pediatric chronic pain.

#### 3.3.2. Parenting

Some studies have focused on parenting factors such as parental reactions to their child’s pain and parental reinforcement of pain behaviors. Protective parenting and solicitous reactions towards their child’s pain behaviors (e.g., reinforcement of pain behaviors, whether intentional or not) are associated with risk of poor adjustment [79,80]. Maternal but not paternal pain catastrophizing is related to pain intensity in children, but neither is related to disability [73]. For children who recently experienced a surgery, reactions by parents who demonstrate more pain catastrophizing within 48–72 h after the surgery predicted greater pain intensity reports in their child 12 months later [41]. One study found that differences in parental bonding style (with the child) were related to rates of chronic pain in children [81]. In addition, the modeling of pain behaviors by parents has also been suggested as a risk factor for youth [82]. Parent protectiveness mediates the relationship between parental cognitions (e.g., pain catastrophizing) and school functioning, including attendance and global ratings of school function [83]. The topic of parent–child interactions represents a promising area of study that may provide fruitful information about the development from acute to chronic pain and/or mechanisms of maintenance.

#### 3.3.3. Family Pain History

Family history of pain has been shown to predict pain in children and adolescents. Children of parents with a specific type of pain are at risk for developing either the same type of pain [8,10,11,50] or other pain-related diagnoses [84,85]. More generally, poor maternal health is related to poor health and function in children with chronic conditions [73]. Family health history is likely to be important in a clinical context, particularly relating to pain, and it appears to be an important avenue for continued research.

#### 3.3.4. Family Environment

A few risk factors related to the family environment have been proposed, including insufficient adult contact [12], single parent household [10], family conflict [86], and low socioeconomic status [18,54]. Disturbances in family functioning have been associated with pain and disability in children [87]. Unexpectedly, family harmony and cohesion has been associated with higher pain in children, although it was hypothesized that the experience of chronic pain in a child may unite families [87]. Anthony and Schanberg [84] posit that family environment plays a crucial role in the experience of juvenile arthritis, although this remains at a theoretical level for this particular disorder. Palermo and Chambers [88] recommend that family communication styles be examined in depth, as little research exists about this particular risk factor. They also highlight the importance of the consideration of family factors in general in a broader model [88].

#### 3.3.5. Social Problems Outside of the Family Context

Poor social functioning has been linked to several chronic pain diagnoses in children and adolescents, including recurrent abdominal pain [36], low back pain [47], fibromyalgia [3], and pain in general [12]. Being bullied is also associated with chronic pain in adolescents [86]. Children with chronic pain often significantly decrease the amount of time spent in activities with peers, including sports and other extracurricular activities [1]. Children who miss significant periods of school due to pain may experience loss of friendships, feel more isolated, are less well-liked, and are less socially accepted than their healthy peers [3,89]. Across a variety of studies, children with chronic pain have reported having fewer friends, were more likely to be victimized by peers, were more isolated, and were evaluated as less likable than healthy peers [90]. In adolescents with chronic pain, pain intensity had a negative impact on independence, emotional adjustment, and identity formation [91].

Perceptions of social functioning also seem to matter. Children who perceived themselves as having poor social competence were more likely to have continued recurrent abdominal pain 5 years later [92]. Adolescents with chronic pain may judge themselves as being less socially developed than their healthy peers [91]. The current research on peer relationships seems to suggest that chronic pain often has a negative impact on the lives of these children and adolescents. However, strong peer relationships may also be protective [91]. Thus, more research on social functioning is needed to provide information about the complex interplay between developmental stage, peer relationships, and pain.

#### 3.3.6. Summary

Research on between-person factors has demonstrated the most support for certain aspects of parenting (e.g., parental response to pain, parental attention), parental psychopathology and family pain history. More research using consistent definitions is needed to fully understand the relationships between family functioning, peer/social functioning, and pediatric chronic pain. The research reviewed here bears similar methodological issues raised in the section on within-person factors. More sophisticated methodology that considers a combination of within- and between-person factors would provide more information about the mechanisms of risk for development of chronic pain to acute pain in children and adolescents.

### 3.4. The Start of a Second Generation of Research

Cummings et al. [93] argue for a second generation of research in child development, particularly relating to childhood psychopathology. The goals of this research are to: (1) identify and understand the causal agents underlying child disorders as dynamic organizations of social, emotional, physiological, genetic, cognitive, and/or other processes; (2) explicate the broader causal net (e.g., multiple processes, risk and protective factors) that accounts for child disorders and the nature of the interrelations between these factors as causal agents; and (3) identify the familial, community, ethnic, cultural, interpersonal, and other contexts that influence causal processes and interrelations between the various dimensions and levels of social contexts [93].

If these goals are applied to pediatric chronic pain research, it appears evident that next steps in research should involve not only larger sample sizes and the replication of outdated studies, but consideration of relationships among risk factors and the utilization of more complex statistical modeling such as group comparison, exploration of mediation and moderation, and hierarchical linear modeling.

Of equal importance, as highlighted by Cummings et al. [93], is the integration of developmental considerations into this second generation of research. Certain stages of development may represent periods of increased susceptibility to either the development or maintenance of chronic pain issues. For example, research demonstrates that children are most vulnerable to the effects of parental depression during specific periods of infancy and adolescence [93]. Such ideas could be extended to chronic pain research in order to determine heightened areas of risk for youth when exposed to significant pain, which could guide preventive and early intervention efforts.

Cummings et al. [93] suggest that, as an individual experiences cumulative successes or failures in stage-salient tasks or developmental transitions, these processes may represent a mechanism by which the individual becomes “stuck” in increasingly stable, diverging trajectories. This is particularly relevant in youth with chronic pain, as some evidence suggests that certain children are at risk for following trajectories with negative prognoses. For example, Mulvaney et al. [53] identified three unique trajectories of recurrent abdominal pain in pediatric patients in a 5-year longitudinal study, including a “long-term risk group”. The long term-risk group did not report the most severe of pain, but had significantly more anxiety, depression, lower perceived self-worth, and more negative life events [53]. Recognition that children face “stage-salient challenges” and incorporation of this information into study designs are crucial for understanding developmental processes and are likely important for understanding the mechanisms that contribute to chronic pain [93].

#### 3.4.1. Examples of Extending the First Generation of Research

One factor that has emerged in the first generation of research on pediatric chronic pain is that of the experience of negative life events [16,52,53,54]. With the presence of negative life events identified as a possible risk factor, we can use this information to explore this risk more deeply. For example, closely related to the experience of stressful life events is one’s ability to cope with that stress. Studies that examine the interaction between the experience of stress and one’s coping style, as measured by physiological response, for example, can provide a deeper understanding of differential responses to pain. Thus, future research that examines the relationships between stressful life events, coping strategies, and pain may provide insight into the mechanisms by which acute pain becomes chronic, and can help to identify targets for treatment. In addition, differences among youth across varying developmental stages could be incorporated into research programs. Understanding the role of development in the transition from acute to chronic pain could potentially shed light on factors that place youth at higher risk for developing chronic pain, as well as those that maintain it.

#### 3.4.2. Prospective, Longitudinal Studies

Another way in which the research on risk factors can carry forward is to design longitudinal studies that examine identified variables over time. Some research groups have already begun to do this by exploring community samples of school children longitudinally in order to understand prevalence of chronic pain. Longitudinal models also allow for the examination of predictor variables that may influence pain later in a child’s life.

One longitudinal study followed children with pain at three time-points across 15 years [94]. At the second phase of data collection, results suggested that youth with pain were more likely than their healthy peers to demonstrate ineffective coping strategies for dealing with stress and to have poor self-esteem [94]. High frequency of nervousness as children predicted pain in adulthood [94]. In addition, 7% of the overall sample reported at the third time point that they were taking antidepressants, and another 3% used sedatives on a regular basis [94]. The incidence of stress reported by the participants also increased over time. Participants attributed their stress to time pressure (38%), occupation (23%), and social relationships (16%). In addition, participants reported significant restlessness (43%), signs of pathological anxiety (34%), and depressive symptoms (13%) [94].

In another study, Walker et al. [95] identified three distinct subtypes of children with recurrent abdominal pain that yielded significant differences in functional disability over time. The three subtypes were labeled high pain dysfunctional, high pain adaptive, and low pain adaptive [95]. The subtypes differed across several characteristics at baseline assessment, including reported levels of abdominal pain, gastrointestinal (GI) and non-GI symptoms, perceptions of threat related to pain, belief of coping ability, levels of pain catastrophizing, negative affect, and health-related impairment. The high pain dysfunctional group appeared to have the greatest of level of difficulty, with the low pain adaptive experiencing the least [95].

At follow-up, the high pain dysfunctional group was characterized by significantly more impairment in a variety of ways. This group was also significantly more likely to meet criteria for a functional GI disorder (FGID) with pain, an FGID with chronic non-abdominal pain, or an FGID with a comorbid anxiety or depressive disorder [95]. In addition, the high pain dysfunctional group showed significantly greater “thermal wind-up” than low pain adaptive patients in laboratory pain testing at follow-up, suggesting greater central nervous system sensitization [95]. Future studies with similar methods, including a physiological component, could provide valuable insight into subtypes of pediatric pain patients with other pain disorders such as complex regional pain syndrome or chronic migraine. Longitudinal study designs such as these are needed to understand causal effects in the transition from acute to chronic pain, as well as maintenance, in children and adolescents.

#### 3.4.3. Mediating Factors

Another important way in which the field can move to a second generation of research is to examine the relationships among various risk factors. Understanding the interactions among variables, using models such as mediation and moderation, will provide more information than single risk factor models. Grunau et al. [96] hypothesize that mother–child interactions may be a mediating factor in children learning to cope with pain. They suggest that sensitive parenting may enhance appropriate interpretation of pain later in childhood for children who are exposed to a great deal of pain earlier in life. Using this theory, transactional processes between parent responses to pain and children’s coping strategies may represent an important process in the transition from acute to chronic pain.

Some studies have moved beyond theory and examined relationships among identified variables. For example, in a community sample of adolescents, anxiety was found to partially mediate the relationship between psychosocial stress and abdominal pain, such that the influence of stress on children’s pain was partially diminished after controlling for anxiety [40]. Involving the family context, Claar et al. [97] found that child anxiety was a moderating factor such that for those with greater anxiety, higher levels of parental protective behavior were associated with higher levels of disability in the child. Child anxiety and depression also separately moderated the relationship between parental pain minimization and children’s somatic symptoms [97].

Packham et al. [58] found that, in combination, five predictors, including, age and pain coping strategies, accounted for 53% of the variance in pain related to pediatric arthritis [58]. By using more sophisticated methodology, such as the interactions among several variables identified in the first generation of research, we can begin to understand the important factors in the mechanisms of the transition from acute to chronic pain. Studies such as this one need to be conducted in pediatric samples. Such advanced modeling provides a context for results of single risk factors from the first generation of research and may provide clues as to the mechanisms of the transition from acute to chronic pain.

#### 3.4.4. Profiles of Response

Sophisticated methodology to determine differential responses to pain or to treatment is another example of research that exemplifies a second generation in research. Walker et al. [95] used hierarchical cluster analyses to determine distinct patterns of coping profiles in pediatric patients with abdominal pain. Six distinct coping profiles were identified: infrequent copers, self-reliant copers, engaged copers, inconsistent copers, avoidant copers, and dependent copers [98]. The method of determining coping strategy profiles involved a 60-item inventory measure that is easily completed by children. An efficient method for categorizing adaptive and maladaptive coping responses in patients was identified, which can be used in clinical settings to help determine treatment goals. For example, if a child relies primarily on poor coping strategies, a greater focus of early treatment could target more effective coping strategies for that child. This research has important implications both for understanding possible mechanisms of the transition from acute to chronic pain as well as maladaptive behaviors that may contribute to maintenance of poor functioning.

## 4. Discussion

This review identified a variety of intra- and interpersonal risk factors that represent the first generation of research on risk for pediatric chronic pain. These studies provide a foundation for a next generation of more sophisticated research on risk mechanisms associated with the transition from acute to chronic pain as well as the maintenance of pain-related functional disability. Several limitations of the current research were identified, including the need for larger sample sizes, multiple formats of measurement (e.g., self- and parent-report, physiological measures), consistent definitions, and replication. Furthermore, longitudinal studies and more complex methodologies are needed to understand temporal and other relationships among these variables.

Importantly, as laid out by the Pediatric Initiative on Methods, Measurement, and Pain Assessment in Clinical Trials (PedIMMPACT) [99], a closer look at functional ability is needed to fully understand mechanisms of chronic pain. The majority of the studies reviewed here consider some characteristic of pain (e.g., presence, severity) as the outcome variable. However, it could be argued that the reason most children and adolescents present for treatment is due to the disability that the pain has caused in their lives, whether it be a decline in school attendance, lessened participation in preferred sports activities, loss of important relationships, and/or other disruptions to day-to-day functioning. Indeed, some researchers have begun following these guidelines in treatment studies, arguing that the primary goal of cognitive-behavioral treatment, a common form of treatment used for chronic pain, is to reduce disability [100].

### *Future Directions* 

Despite the problems listed, a first generation of research is needed to provide the basis for the next generation. In other words, the studies reviewed here are a necessary step to identify variables that can then be explored in a more complex manner. A second generation of research must then be generated to elucidate knowledge gained from the first generation [101]. A second generation would include efforts in two main areas: (1) improvement from the existing research, including replication with larger sample sizes, the addition of physiology and developmental stage into conceptual models, and further research in family and peer factors related to the development of chronic pain, and (2) more sophisticated methodological approaches, including the use of comparison studies, examination of interactions among previously identified variables, use of longitudinal design, and analyses such as hierarchical linear modeling and cluster analyses to determine bidirectional influences and trajectories, respectively. Hierarchical linear modeling can be used to account for dynamic interactions between two individuals (e.g., parent–child) and changes over time (e.g., different developmental stages, actor–partner influence in real time) [102]. Finally, once the second generation is complete, a third generation of research may be needed to identify causal relationships [103].

A biopsychosocial perspective in a developmental context is proposed (see Figure 1), which would extend beyond the limitations of the first generation of research. In addition to the risk factors identified thus far, we first propose that interactions among these factors be explored in relation to the development of chronic pain.

The “biological” piece of biopsychosocial considerations has not received as much attention in the literature. In the context of mediating and moderating relationships, the youth’s own physiological functioning may be an important area of consideration. For example, respiratory sinus arrhythmia, or heart rate variability, as well as skin conductance are promising areas of research. These underlying physiological processes may further complete the picture of the transition from acute to chronic pain. Respiratory sinus arrhythmia is thought to be an index of emotion regulation on a physiological level [104,105], which may play a role in children’s and parents’ responses to chronic pain. Indeed, a study of adults with fibromyalgia and temporomandibular disorder found that patients demonstrated greater changes in respiratory sinus arrhythmia than controls, which was thought to reflect hyperarousal in an inappropriate context [106]. Other examples of biological contributors or processes that have been associated with pediatric chronic pain include visceral sensitivity [107,108] seen in children who have recurrent abdominal pain and abnormal pain signaling in complex regional pain [109].

Furthermore, in the treatment literature, a number of studies assess the efficacy of biofeedback for children and adolescents with chronic pain. Biofeedback typically involves therapist-assisted measurement and observation of physiology as well as suggestions for improvement from the therapist [110]. Several studies have demonstrated promising results in treatment of youth with biofeedback procedures [111,112], suggesting the physiology plays an important role in chronic pain and may be modified to improve the quality of life of individuals dealing with chronic pain. Underlying physiological mechanisms, particularly in the context of chronic pain disorders, which are very closely tied to medical issues, would be a valuable addition to Palermo and Chambers’ [88] model.

Regarding the “social” piece of biopsychosocial considerations, in addition to inclusion of family and/or parent factors, peer relationships require further examination. Peer relationships or social functioning may play a role in the transition from acute to chronic pain, as many pediatric patients are likely to be in a stage of adolescence where peer relationships are incredibly salient. This area of focus also touches upon the developmental framework proposed, as the “tasks” of development differ depending upon age and stage of development. For example, adolescence marks an important time for development of significant peer relationships and identity formation. Certain aspects of peer relationships, such as the quality of these relationships, social support, victimization, and other salient elements could be included in mediation and moderation models. Coping strategies, thought to change across childhood, are another area of potentially fruitful, developmentally oriented research. Thus, development is an important context that must be included in consideration of risk in pediatric pain.

The “psychological” piece of the biopsychosocial model has perhaps received the most attention in the literature. The proposed next steps integrate all aspects of the biopsychological model, as well as developmental considerations. This proposal extends beyond Palermo and Chamber’s [88] important model by adding psychophysiological, peer, and developmental variables (see Figure 1). Furthermore, complex methodology can provide a more thorough understanding of the interactions among variables related to the development, and maintenance, of pediatric chronic pain. These next steps represent the second generation of research in pediatric chronic pain, which can inform prevention and early intervention efforts.
